# Parental acceptance of typhoid conjugate vaccine for children aged 6 months to 15 years in an outbreak setting of Lyari Town Karachi, Pakistan

**DOI:** 10.1016/j.vaccine.2023.07.003

**Published:** 2023-08-23

**Authors:** Rabab Batool, Mohammad Tahir Yousafzai, Sonia Qureshi, Sajid Muhammad, Ibtisam Qazi, Tahira Sadaf, Per Ashorn, Farah Naz Qamar

**Affiliations:** a Department of Pediatrics and Child Health, Aga Khan University Hospital, Stadium Rd, PO Box 3500 Karachi 74800, Pakistan; b Centre for Child, Adolescent, and Maternal Health Research, Faculty of Medicine and Health Technology, Tampere University, Arvo Ylpön katu 34, Tampere 33520, Finland; c The Kirby Institute, UNSW Sydney, Wallace Wurth Building High Street, UNSW Sydney, Kensington, NSW 2052, Australia

**Keywords:** Outbreak, Typhoid conjugate vaccine, Cross-sectional study, Parental acceptance, Vaccine attitudes scale

## Abstract

**Background:**

This study aimed to evaluate the parental acceptance of Typhoid Conjugate Vaccine (TCV) and to determine the predictors of TCV vaccination status among children in an outbreak setting of extensively drug resistant (XDR) typhoid fever in Karachi, Pakistan.

**Methods:**

A cross-sectional survey using the WHO recommended rapid vaccine coverage assessment technique was conducted. Out of 11, four union councils (UCs) in Lyari Town were randomly selected. A parent or primary caretaker from the eligible household was interviewed. Data were collected using a locally validated vaccine attitudes scale (VAS). Sum of scores was calculated for VAS. A higher score denoted negative attitudes and perceptions regarding TCV and vice versa. Multivariable logistic regression was performed to determine the predictors of TCV vaccination status.

**Results:**

Based on the 14-item parental VAS, 78.0 % of the parents had a score between 0 to <40 and 22 % had a score ≥40. VAS score of <40 was significantly associated with higher odds of receiving TCV during the campaign setting (adjusted Odds Ratio (aOR): 1.30; 95 % Confidence Interval (CI): 1.02, 1.66). The odds of receiving TCV vaccination were higher among children whose parents were aware of the ongoing vaccination campaign in the area (aOR: 4.57; 95 % CI: 2.93, 7.12) and expressed willingness to get their child vaccinated against typhoid fever (aOR: 2.54; 95 % CI: 1.82, 3.55).

**Conclusion:**

Parental awareness of the ongoing vaccination campaign, positive perception and attitudes towards vaccine were found to be significantly associated with TCV vaccination among children. Appropriately structured pre-vaccination awareness campaigns focused on childhood vaccination targeted towards parents are necessary to improve parental awareness, attitude and behavior towards vaccination.

## 1 Introduction

The emergence and spread of antimicrobial drug resistance (AMR) has reduced the efficacy of antibiotics for treating typhoid fever. This has led to a resurgence of this disease especially in the South Asian, Southeast Asian, and sub-Saharan African regions [1]. An outbreak of extensively drug resistant (XDR) typhoid was reported in Hyderabad, Pakistan in the last quarter of 2016 [2]. The circulating strain of XDR belonged to the H58 haplotype, which is common in parts of Asia and Africa and resistant to the first-line antibiotics used for treating typhoid fever such as chloramphenicol, ampicillin, trimethoprim-sulfamethoxazole, and ciprofloxacin [3]. It was also resistant to ceftriaxone, which was frequently used for the empirical treatment of this disease [2]. Widespread AMR is associated with a higher rate of complications and deaths, as well as prolonged fecal shedding, which sustains transmission and induces secondary cases. Novel therapies to treat resistant strains and primary preventive measures such as vaccines [4] have a major role in controlling the emergence and spread of this disease [5].

In December 2017, World Health Organization (WHO) approved the Typhoid Conjugate Vaccine (TCV) for use in typhoid endemic regions and epidemic settings [5]. The introduction of TCV in 2019 through GAVI, The Vaccine Alliance (GAVI) in low- and middle-income typhoid endemic countries ensured increased access through vaccination campaigns [6]. Despite the increased accessibility, vaccine hesitancy was a concern for the uptake of this vaccine [7]. We conducted a cross-sectional survey of parents of children aged 6 months to 15 years residing in Lyari Town Karachi, after the completion of the vaccination campaign to evaluate parental acceptance of TCV and to determine the predictors of TCV vaccination status among children in an outbreak setting of XDR typhoid.

## 2 Materials and methods

### 2.1 Study design and setting

A cross-sectional survey was conducted from 8th to 18th November 2019 in Lyari Town, Karachi using the WHO recommended rapid vaccine coverage assessment technique (30 clusters & 7 households per cluster) [8]. Lyari Town is one of the 18 towns located in Karachi, Pakistan. The total population of Lyari is approximately 6.6 million. During 2019, there was an outbreak of XDR typhoid in Karachi and as a response to the outbreak Aga Khan University (AKU) launched a mass immunization campaign in Lyari Town, which was one of the epicenters of the XDR typhoid outbreak. The vaccination campaign started from 10th April 2019 and ended on 25th October 2019, inoculated 87,993 children aged 6 months to 15 years with a single shot of TCV (Typbar TCV® manufactured by Bharat Biotech) and had an estimated coverage of 80 %. This survey was conducted immediately after the vaccination campaign.

### 2.2 Study population

The target population included parents or caregivers with at least one child aged 6 months to 15 years of age (eligible to receive a dose of TCV during the campaign) who resided in Lyari Town and were willing to provide written informed consent to participate in the survey.

### 2.3 TCV vaccination exposure assessment

Data on the TCV immunization status of children aged 6 months to 15 years were collected immediately after the completion of the TCV campaign to limit recall bias. Vaccination status was considered positive only if the child had been vaccinated in the AKU-led TCV immunization campaign and was verified through vaccination campaign registries. Photographs of vaccination cards were taken to verify the vaccination status and formed part of the data record.

### 2.4 Sampling technique and sample size calculation

The WHO recommended rapid vaccine coverage assessment technique (30 clusters & 7 households per cluster) was used [8]. There are 11 Union Councils (UCs); the lowest level of administrative unit, in Lyari Town. Four UCs were randomly selected using simple random sampling without replacement. Each UC was further divided into clusters and 30 clusters were randomly selected from each of the four UCs. The number of households in each cluster was between 200 and 250. The spatial sampling tool was used to generate seven random sample points for the selection of houses in each cluster. Using this strategy, we included 210 households from each UC with a total number of 840 households being included in this survey.

A trained team of research assistants accompanied by people from the local community reached the randomly selected households using live maps on tablets for navigation to reach the correct household. After obtaining written informed consent, data were collected through face to face interviews. Upon identification of the household, it was assessed for eligibility to participate in the survey otherwise, the next household was approached. At least, one parent/or primary caregiver from each household (whoever was present in the household at the time of survey) was interviewed. Each interview was of approximately 20 minutes in duration. If the parent/caregiver was busy or not available for the interview, the team revisited the household at another suitable time to conduct the interview.

### 2.5 Data collection tools and process

The acceptability of TCV vaccination was assessed using a locally validated parental vaccine attitudes scale (VAS) [9]. The VAS is a 14-item Likert scale. The scale has two subdomains: one for vaccine perceptions and concerns (10 items) and the other for disease salience and community benefits (4 items). Each item on the VAS has a 5-point scale with responses ranging from strongly agree to strongly disagree (1 = strongly agree, 2 = agree, 3 = not sure, 4 = disagree, and 5 = strongly disagree) and a total possible score ranging from 14 to 70. Eight Likert scale items from the total are structured in a way that the numerical scoring scale runs in the opposite direction, hence reverse coding of these items was performed to calculate the total sum of score for VAS.

The questionnaire including VAS comprised of TCV vaccination status, age of the child, place of immunization, time to reach the vaccination center, date of vaccination and information on parental experience, exposure, attitude, knowledge and perception regarding the TCV vaccine. The questionnaire was translated into the local Urdu language and all the interviews were conducted in Urdu. We developed an electronic data capture program for real-time data collection. Data were synced to a central server at the Aga Khan University.

### 2.6 Data quality and analysis

Random spot observations of interviews and re-interviews of 10 % of the recruited households were conducted to maintain data quality. The data were sent to the central data management unit (DMU) in the Department of Pediatrics at Aga Khan University Karachi on a daily basis.

STATA version 16.0. was used to analyze the data. We calculated the sum of scores for the 14-item VAS, the average of the sum of scores for the VAS and sub scales were broken into two categories moderate to high and low based on the defined cutoff points. “Low” scores were defined as a score of <40 on the VAS, <30 on the vaccine perceptions and concerns subscale and between 4 and 12 on the disease salience and community benefit subscale [9]. To identify the determinants of typhoid vaccination status multivariable logistic regression was done to determine the associations between parental personal typhoid disease experience, attitude, knowledge and perceptions regarding the vaccine and vaccination status of their children. We used logistic regression to assess the association between scores on VAS subscales and vaccination status of the children. Variables with a p-value of <0.25 in univariate analysis were included in the multivariable model. Best subset method was used to develop the final model. Homser and Lemeshow goodness of fit test was used to assess the final model adequacy. All analyses are based on two-sided p-values, with statistical significance defined by p-value of less than <0.05.

## 3 Results

A total of 840 households were approached, of which 15 (6.5 %) were ineligible (did not have children aged 6 months to 15 years) and 5 (2.2 %) refused to participate in the vaccine coverage and acceptability survey. All the households which agreed to participate in the vaccine coverage survey were offered the VAS in addition to the vaccine coverage survey.

A total of 2325 children aged 6 months to 15 years were analyzed for vaccination status. Out of these 1857 (79.9 %) of children had received the TCV vaccine in the campaign, 1163 (50.1 %) were male and the mean age of the children was 7.6 years (Standard Deviation (SD) ± 3.8). Only 978 (52.7 %) of the parents of the vaccinated children provided vaccination cards. Vaccination status of the children whose parents could not provide the vaccination cards, was confirmed from vaccination records of the healthcare registers.

A total of 875 (47.1 %) children received their TCV vaccination at their respective schools. The details of the sites of vaccination for all children who received a single shot of TCV during the vaccination campaign are given in Table 1.

**Table 1:** Demographic characteristics of the children included in the study (N = 2325)

Characteristics	N (%)
*Gender*	
Male	1163 (50.1)

*Age groups*	
<5 years	585 (25.2)
5–9 years	957 (41.2)
10–15 years	783 (33.6)
Mean age ± SD (years)	7.6 ± 3.8

*Relationship of respondent with the child*	
Parent	2236 (96.2)
Primary caregiver	33 (1.4)
Others	56 (2.4)
*Child vaccinated against typhoid fever*	1857 (79.9)
*Possession of TCV vaccination card at the time of interview*	978 (52.7)

*Site where TCV was administered*	
Kharadar General Hospital	29 (1.6)
Lyari General Hospital	118 (6.3)
Aga Khan Secondary Care Hospital Kharadar	146 (7.9)
School	875 (47.1)
Others*	689 (37.1)

*Mode of transport to the health facility*	
By car/bike	252 (13.6)
Taxi/auto rickshaw	159 (8.6)
Public transport bus/minibus	25 (1.3)
On foot	1421 (76.5)

*Time to reach preferred vaccination center*	
0–14 minutes	1228 (66.1)
15–30 minutes	617 (33.2)
>30 minutes	12 (0.7)
Mean time ± SD (minutes)	11.5 ± 8.0

* Others category refers to the community-based vaccination campaign and mop-up activity at schools.

Parents were asked about their personal experiences regarding typhoid disease and 836 (36.0 %) of the interview respondents had personally seen someone with typhoid fever and 543 (23.4 %) of them knew someone who had experienced typhoid in their family or community. When parents were asked if they had ever delayed the vaccination of their children, only 31 (1.3 %) of the parents mentioned that they had delayed their child’s vaccination for reasons other than illness or allergy. Majority of parents, 2143 (92.2 %) showed willingness to get their child vaccinated with typhoid vaccine, 593 (25.5 %) of the parents were aware of the location of the vaccination centers and 901 (38.8 %) of them were aware of the service timings of those vaccination centers.

We found that 1122 (48.3 %) parents believed that the vaccine could cause fever, 947 (40.7 %) mentioned that pain at the site of injection might occur, 279 (12.0 %) mentioned that pustules could develop at the site of the injection and 574 (24.7 %) mentioned that vaccine could cause irritability. Among the parents surveyed, a small proportion, 254 (10.9 %) mentioned that the vaccine could cause disturbed sleep and 54 (2.3 %) had a perception that vaccine could cause improper feeding behavior in children (Table 2).

**Table 2:** Parental personal typhoid disease experience, exposure, attitude, knowledge and perception regarding vaccination (N = 2325)

	N (%)
Personally seen someone with typhoid fever	836 (36.0)
Knew someone in the family or community who had typhoid disease	543 (23.4)
Delayed having child get a vaccine for reasons other than illness or allergy (n = 1857)	31 (1.3)
Had ever decided not to have the child get a vaccine	28 (1.2)
Willingness to get the child vaccinated with typhoid vaccine	2,143 (92.2)
Awareness regarding the location of vaccination services	593 (25.5)
Awareness regarding the time when vaccination services were offered	901 (38.8)

Parental perception regarding the side effects of vaccination among children	
Fever	1,122 (48.3)
Pain at site of injection	947 (40.7)
Pustule	279 (12.0)
Irritability	574 (24.7)
Disturbed sleep	254 (10.9)
Inappropriate feeding behavior	54 (2.3)
Others	5 (0.2)

Nearly two-thirds (61.1 %) of the parents mentioned that children are getting more vaccines than the number of vaccines actually good for them while 313 (13.4 %) of the parents believed healthy children do not need vaccination. Furthermore, 829 (35.7 %) of the parents surveyed strongly agreed or agreed that it is better for their child to develop immunity by getting sick than through vaccination. Most of the parents (2100 (90.3 %)) thought that they should be allowed to selectively choose the vaccines which they believe their child needs (Table 3).

**Table 3:** Descriptive statistics of individual items of parental vaccine attitudes scale (N = 2325)

Item on VAS	N (%)
*Children get more vaccinations than actually good for them*	
Strongly agree	305 (13.1)
Agree	1116 (48.0)
Not sure	151 (6.5)
Disagree	713 (30.7)
Strongly disagree	40 (1.7)

*Healthy children do not need vaccinations*	
Strongly agree	103 (4.4)
Agree	210 (9.0)
Not sure	137 (5.9)
Disagree	1635 (70.3)
Strongly disagree	240 (10.3)

*Vaccinations do more harm than good*	
Strongly agree	34 (1.5)
Agree	108 (4.6)
Not sure	245 (10.5)
Disagree	1641 (70.6)
Strongly disagree	297 (12.8)

*It is better for my child to develop immunity by getting sick than to get a vaccination*	
Strongly agree	55 (2.4)
Agree	774 (33.3)
Not sure	685 (29.5)
Disagree	771 (33.2)
Strongly disagree	40 (1.7)

*I should be allowed to selectively choose the vaccines which I believe my child needs*	
Strongly agree	565 (24.3)
Agree	1535 (66.0)
Not sure	68 (2.9)
Disagree	141 (6.1)
Strongly disagree	16 (0.7)

*It is better for my child to receive two injectable vaccines in one visit rather than one injectable vaccine in two visits*	
Strongly agree	112 (4.8)
Agree	921 (39.6)
Not sure	249 (10.7)
Disagree	881 (37.9)
Strongly disagree	162 (7.0)

*I believe many of the illnesses which vaccines prevent are severe*	
Strongly agree	357 (15.4)
Agree	1558 (67.0)
Not sure	331 (14.2)
Disagree	74 (3.2)
Strongly disagree	5 (0.2)

*When I see a child or a picture of a child with either Polio, Diphtheria, Pertussis (whooping cough), tetanus, hepatitis B, pneumonia, meningitis or measles, I am reminded of the need for vaccination*	
Strongly agree	673 (28.9)
Agree	1454 (62.5)
Not sure	92 (4.0)
Disagree	106 (4.6)

*When my child is vaccinated, it benefits my entire community by reducing the spread of disease*	
Strongly agree	151 (6.5)
Agree	1539 (66.2)
Not sure	450 (19.4)
Disagree	156 (6.7)
Strongly disagree	29 (1.2)

*When a parent refuses to vaccinate a child, it harms the entire community through risk of disease*	
Strongly agree	117 (5.0)
Agree	1151 (49.5)
Not sure	750 (32.3)
Disagree	280 (12.0)
Strongly disagree	27 (1.2)

*I am concerned my child might have a serious side effect from vaccination*	
Strongly agree	25 (1.1)
Agree	479 (20.6)
Not sure	328 (14.1)
Disagree	1450 (62.4)
Strongly disagree	43 (1.8)

*Following the vaccination schedule is a good idea for my child*	
Strongly agree	645 (27.7)
Agree	1635 (70.3)
Not sure	25 (1.1)
Disagree	15 (0.6)
Strongly disagree	5 (0.2)

*Parents who do not vaccinate their children should be penalized with a monetary fine*	
Strongly agree	287 (12.3)
Agree	814 (35.0)
Not sure	518 (22.3)
Disagree	625 (26.9)
Strongly disagree	81 (3.5)

*I would like to be a volunteer advocate for vaccination in my community if I am trained*	
Strongly agree	375 (16.1)
Agree	1275 (54.8)
Not sure	319 (13.7)
Disagree	330 (14.2)
Strongly disagree	26 (1.1)

Children whose parents were aware of the ongoing vaccination campaign in their area had a higher odds of being vaccinated (adjusted Odds Ratio (aOR): 4.57; 95 % Confidence Interval (CI): 2.93, 7.12) as compared to the children whose parents were unaware of the vaccination campaign in their area. The odds of being vaccinated were higher (aOR: 1.21; 95 % CI: 0.97, 1.52) among children whose parents had personally seen someone with typhoid fever as compared to those who had not observed someone suffering from typhoid fever. Children of parents who showed willingness to get their child vaccinated for typhoid fever had higher odds of being vaccinated (aOR: 2.54; 95 % CI: 1.82, 3.55) as compared to the children of hesitant parents. Parental knowledge regarding the timings of the vaccination services being offered at vaccination centers was significantly associated with higher odds (aOR: 1.92; 95 % CI: 1.45, 2.54) of their child being vaccinated. Parental perception of side effects specifically pustule formation among children was associated with a positive TCV vaccination status of children as compared to the opposing perception that vaccination does not cause side effects among children (aOR: 1.52; 95 % CI: 1.06, 2.18). A low VAS score among parents was significantly associated with higher odds (aOR: 1.30; 95 % CI: 1.02, 1.66) of their children being vaccinated as compared to a high VAS score (Table 4).

**Table 4:** Association of parental personal typhoid disease experience, exposure, attitude, knowledge and perception regarding vaccine with the TCV vaccination status of children

	Not vaccinated (N = 468)	Vaccinated (N = 1857)	Unadjusted OR (95 % CI)	Adjusted OR (95 % CI)
	N (%)	N (%)		
*Are you aware of ongoing typhoid vaccination campaign in your area?*				
Yes	418 (49.3)	1813 (97.6)	4.93 (3.24, 7.49)	4.57 (2.93, 7.12)
No	50 (10.7)	44 (2.4)	Ref.	Ref.

*Have you personally seen someone with typhoid?*				
Yes	148 (31.6)	688 (37.1)	1.27 (1.02, 1.58)	1.21 (0.97, 1.52)
No	320 (68.4)	1169 (62.9)	Ref.	Ref.

*Do you know of someone in your family or community who had typhoid?*				
Yes	109 (23.3)	434 (23.4)	1.00 (0.79, 1.28)	
No	359 (76.7)	1423 (76.6)	Ref.	

*Would you want your child to get typhoid vaccination?*				
Yes	398 (85.1)	1745 (94.0)	2.74 (1.99, 3.77)	2.54 (1.82, 3.55)
No	70 (14.9)	112 (6.0)	Ref.	Ref.

*Have you ever decided not to have your child vaccinated?*				
Yes	25 (5.3)	3 (0.2)	–	–
No	443 (94.7)	1854 (99.8)		

*Do you know the location of vaccination services?*				
Yes	317 (67.7)	1415 (76.2)	1.52 (1.22, 1.90)	0.75 (0.55, 1.03)
No	151 (32.3)	442 (23.8)	Ref.	Ref.

*Do you know the time when vaccination services are offered?*				
Yes	235 (50.2)	1189 (64.1)	1.76 (1.44, 2.17)	1.92 (1.45, 2.54)
No	233 (49.8)	668 (35.9)	Ref.	Ref.

*What do you think are the side effects of vaccination among children?*				
*Fever*	241 (51.5)	962 (51.8)	0.99 (0.81, 1.21)	
Yes	227 (48.5)	895 (48.2)	Ref.	
No				

*Pain at site of injection*				
Yes	196 (41.9)	751 (40.4)	0.94 (0.77, 1.16)	
No	272 (58.1)	1106 (59.6)	Ref.	

*Pustule*				
Yes	40 (8.6)	239 (12.9)	1.58 (1.11, 2.25)	1.52 (1.06, 2.18)
No	428 (91.4)	1618 (87.1)	Ref.	Ref.

*Irritability*				
Yes	116 (24.8)	458 (24.7)	0.99 (0.79, 1.26)	
No	352 (75.2)	1399 (75.3)	Ref.	

*Disturbed sleep*				
Yes	49 (10.5)	205 (11.0)	1.06 (0.76, 1.48)	
No	419 (89.5)	1652 (89.0)	Ref.	

*Inappropriate feeding behavior*				
Yes	16 (3.4)	38 (2.0)	0.59 (0.33, 1.07)	0.68 (0.36, 1.26)
No	452 (96.6)	1819 (98.0)	Ref.	Ref.

*Others*				
Yes	5 (1.1)	0 (0.0)	–	–
No	463 (98.9)	1,857 (100.0)		

*VAS score*				
Low (<40)	345 (73.7)	1469 (79.1)	1.35 (1.07, 1.71)	1.30 (1.02, 1.66)
Moderate to high (40–70)	123 (26.3)	388 (20.9)	Ref.	Ref.

OR: odds ratio. CI: confidence interval. Ref. : reference category. All potential covariates (p-values < 0.25 in the univariate analysis) were included in the multivariable analysis and dropped consecutively based on statistical significance. The variables having p-value < 0.05 were retained in the model for final adjustment.

In the adjusted model, after controlling for age and gender parents who scored low on the overall 14-item VAS scale had higher odds (aOR: 1.39; 95 % CI: 1.09, 1.76) of getting their children vaccinated as compared to their counterparts. Likewise, parents who scored low on the 10-item vaccine perceptions and concerns scale had higher odds (aOR: 1.49; 95 % CI: 1.18, 1.87) of getting their children vaccinated as compared to the parents who scored moderate to high. Low score on 4-item attitude scale towards disease salience and community benefit was significantly associated with higher odds (aOR: 1.44; 95 % CI: 1.09, 1.91) of children being vaccinated as compared to a moderate to high score (Table 5).

**Table 5:** Association of parental VAS score with the immunization status of children

Scale	N (%)	Unadjusted OR (95 % CI)	Adjusted OR (95 % CI)
*VAS*			
Low (<40)	1814 (78.0)	1.35 (1.07, 1.71)	1.39 (1.09, 1.76)
Moderate to high (40–70)	511 (22.0)	Ref.	Ref.

*Vaccine perception and concerns scale*			
Low (<30)	752 (32.3)	1.40 (1.12, 1.75)	1.49 (1.18, 1.87)
Moderate to high (30–50)	1573 (67.7)	Ref.	Ref.

*Attitude towards disease salience and community benefit scale*			
Low (4–12)	2019 (86.8)	1.43 (1.08, 1.89)	1.44 (1.09, 1.91)
Moderate to high (13–20)	306 (13.2)	Ref.	Ref.

OR: odds ratio. CI: confidence interval. Ref. : reference category. Outcome variable: child immunization status (0 = un-immunized, 1 = immunized). Multiple linear regression model adjusted for age and gender of the child.

## 4 Discussion

This is the first study to report parental acceptance of TCV introduced for the prevention of typhoid in an outbreak setting of Karachi, Pakistan. Parental awareness regarding the ongoing vaccination campaigns in their area of residence, the time at which vaccination services are offered at the vaccination centers, having personally observed someone with typhoid, parental willingness to get their children vaccinated, perception regarding side effects and a low score on VAS scale were the factors found to be significantly associated with TCV vaccine uptake among children in the outbreak setting of Lyari Town Karachi, Pakistan.

We found that parents of unvaccinated children were not even aware of the ongoing typhoid vaccination campaign in their area and the timings of services offered at the vaccination centers. Awareness campaigns in low coverage areas may help to minimize disease spread by improving vaccination uptake [10]. Previous studies have also reported that in urban settings culturally appropriate social mobilization activities to sensitize the targeted population to immunization can increase parental awareness, intention to immunize children and eventually improve vaccine uptake [11]. Recent studies on the COVID-19 vaccine have also reported that vaccine hesitancy was closely associated with awareness, education and source of information in Africa [12]. A pre-vaccination campaign for increasing community awareness should be focused on promoting the benefits of vaccination at the individual and community levels. These campaigns should convey a message regarding the upcoming immunization campaign, the location and timings of vaccination at nearby health facilities and address misconceptions about the vaccines before they become widespread.

In our study, parents who had personally observed someone with typhoid were more likely to get their child vaccinated. Research has shown that vaccination decision-making should be considered and understood in a wider socio-cultural context as vaccination is part of a “wider social world” and decision making for vaccination is highly influenced by various social factors including past experiences with health services, family history and community experiences with vaccination [13].

This study showed that parents’ favorable attitudes towards vaccinating children against typhoid fever are significantly associated with their children’s vaccination status for TCV. Children of the parents who expressed their willingness to get their child vaccinated with TCV had higher odds of being vaccinated. The results of our study are concordant with other studies which also reported that a positive parental attitude towards vaccination was found to be associated with the vaccination status of their children [14], [15]. A positive attitude towards vaccination was seen among parents who were aware of influenza vaccines in Pakistan [16]. Therefore, appropriately structured awareness programs about childhood vaccination targeted towards parents are necessary to improve parental attitude and behavior towards vaccination.

Parents who mentioned that the vaccine could cause pustule formation at the injection site were more likely to get their child vaccinated as it is a common perception in slum areas that experiencing side effects after getting vaccinated means the vaccine is working.

We have found that the parents who scored low on the VAS scale, which represents positive attitudes and perceptions of the parents toward childhood vaccination, were more likely to get their children vaccinated. The results of the study are concordant with a previous study conducted in the similar setting where the parents of the unimmunized or partially immunized children were more likely to score high on the overall 14-item VAS scale, 10-item vaccine perceptions and concerns subscale, and 4-item disease salience and community benefit subscale as compared with the parents of fully immunized children [9].

Majority of the parents preferred school or a private setting for the vaccination. Since most parents walked to the vaccination center, having mobile vaccination camps can improve vaccination coverage [17]. The data reflect the ease of access to the TCV vaccine, which was due to the mass immunization campaign in an emergency context therefore the impact of geographic barriers on parental intent to vaccinate their children could not be assessed. A study conducted in Pakistan reported that the distance of the vaccination center impacts a child’s vaccination status and the proportion of unvaccinated children increased when the distance to the vaccination center was increased [18].

The majority of the parents agreed that they would like to be volunteer advocates for vaccination in their community if they can get appropriate training. Moreover, most of the respondents agreed that the existing vaccination schedule is good for their children and vaccines prevent severe diseases. However, some parents had concerns regarding the number of vaccines their children receive, and a significant number of parents agreed that they should be given the option to selectively choose the vaccine for their children that they believe their child needs, with a large number of the parents agreeing with the idea that the children receive more vaccines than they actually need. The perception of parents that children receive more vaccines now than previously could affect parental vaccine hesitancy for new vaccines [19], [20]. A significant number of respondents had the opinion that it is better for the child to develop immunity by getting sick than to get a vaccine, while 80 % of parents mentioned that they should be allowed to selectively choose the vaccines which they believe their children need. Attitudes towards vaccines cannot be polarized into anti-vaccine or pro-vaccine as previously thought but fall in a continuum, ranging from full acceptance to outright refusal for some or all vaccines. This is a complex phenomenon and vaccine-specific issues must be understood contextually and conceptually. We recommend that more research be undertaken to explore effective strategies that can combat the issue of vaccine hesitancy in our study population [3].

## 5 Conclusion

Parental awareness of the ongoing vaccination campaign, personally seeing someone with typhoid fever, willingness to get the child vaccinated, knowledge of the location and timings of the vaccination services and a lower VAS score are found to be significantly associated with TCV vaccination among children. Adequate promotional activities to improve awareness about the vaccination campaign, and targeting parents with higher VAS score are recommended to improve vaccine acceptance during the outbreak setting.

## Ethics approval and consent to participate

The study received approval from the Ethical Review Committee (ERC) of Aga Khan University, Karachi, Pakistan as part of a larger study assessing the impact of Typhoid Conjugate Vaccine (#0887). Written consent for study participation was obtained from parents/caregivers of all study participants. Participation in this survey was voluntary and participants were not identified on the questionnaire. Each household was given a unique identification code to maintain confidentiality.

## Funding

This research was funded by the Bill and Melinda Gates Foundation, grant number INV000640_2018. The funding agency had no role in the design of the study, data collection, analysis, interpretation of data or in writing the manuscript.

## Contributions

F.N.Q, R.B and M.T.Y conceived, designed and executed the study. R.B supervised data collection. M.A and T.S were responsible for study coordination. R.B conducted qualitative analysis and developed the implementation framework. R.B and S.A conducted quantitative analysis. R.B, F.N.Q and M.T.Y discussed results and interpretation. R.B drafted the manuscript with contributions from F.N.Q and M.T.Y. All authors read and approved the final manuscript.

## Declaration of Competing Interest

The authors declare that they have no known competing financial interests or personal relationships that could have appeared to influence the work reported in this paper.

## Data Availability

Data will be made available on request.
